# Six Year Molars

**Published:** 1864-06

**Authors:** Frank Abbott


### SIX YEAR MOLARS.

Read before the Brooklyn Dental Association.

BY FRANK ABBOTT.

I do not expect, Mr. President, to give you or any member of the Association present any new idea in relation to the treatment of these teeth, but to do what I can toward sustaining the position I have already taken; which is, as the most of you are aware, that these teeth should never be extracted.

What I wish to be understood by the word *never* is, that they should never be sacrificed for any cause which is not sufficient for the extraction of any other teeth in the mouth. In other words, they should be preserved in the mouth with as much care as the incisors, twelve year molars, or any others.

I know they usually come to us in the mouths of young and nervous children, and that it requires a great deal of patience and often a great amount of labor to make fillings sufficient for their preservation, and but for claims our patients have upon us as Dentists, and honest men (as we all ought to be), we would extract a large number of them to get rid of such troublesome patients.

I know they are almost universally neglected by parents, which we attribute wholly to their ignorance, and which is the main cause of so much suffering and loss of these valuable organs, and until we do something to educate the people, as they should be on this subject, we will find about the same state of things that we have always found. These six year molars, with their crowns half, two-thirds, and, perhaps, entirely gone, before they are taken to a dentist. I seem to hear some one ask, What would you do if you found them in this last condition? I would, perhaps, extract them. It would depend altogether upon the state of the surroundings, generally speaking; if healthy, I would let them remain, and do what I could to preserve them, until the jaw was developed to my satisfaction. If diseased and in a highly inflamed and ulcerating condition, I would usually consider the patient's mouth in a better condition with them out than in. Teeth and roots of teeth are very different. Some dentists give as a reason why they should be extracted almost indiscriminately, that those remaining may have room to spread apart, and, as they say, be less liable to decay from the retention and decomposition of food.

The fact is, teeth will not decay from this cause, no matter how near they are together, if kept properly cleaned. No, the nearer the better, for every tooth should have support from its neighbor to protect it in mastication.

It is often that teeth standing alone become very sore, from biting some hard substance to a disadvantage, and sometimes the periosteum becomes so much inflamed that the death of the palp is the result. This you never find where teeth support each other as they should. Like our Wall Street financiers, when hard pressed, they always look to their friends for support; if on hand with strength sufficient, all right; if not, they most go by the board. We are all dependent upon each other for our life, living and general well-being, and no more so than each tooth is dependent upon another.

I should not feel very well pleased if I had been taken to a dentist, when young, and had my six year molars extracted, because my teeth happened to be very close together, although I have seventeen teeth in my under jaw. Now if I had accidentally fallen into the hands of some one of these not "modern dentists," but one of those who claim teeth should stand alone, my jaws would probably be considerably reduced in size from what they are now. Gentlemen, I need all the teeth I have, and since I have heard this doctrine of extracting six year molars, so almost universal, I have thanked God that there were no dentists in the section of country where I spent the earlier part of my life.

Did nature intend that these teeth should be sacrificed so early? The largest, strongest and, as it would seem, were intended to be of the greatest amount of service of any teeth in the mouth. I ask, did nature place these teeth in the mouth, in this high state of development, to be taken out as soon as they are through the gum far enough to get a good hold of? I certainly think not.

We hear it asked, did nature intend that teeth should decay or come in irregular? No; this, as our friend Dr. Atkinson would say, is the work of the devils, and I consider it the duty of every honest man to do all in his power toward arresting and correcting such works, and in the best possible manner. If teeth decay, arrest it by properly filling them. If they come irregular, regulate them, not by extracting six year molars, but by spreading the mouth, making it large enough to hold all the teeth perfectly. There is seldom any danger of making it too large. If children were taken to good dentists as early as they ought to be, which should be when they are from two to three years old, and as often after as the cases require, we would find but very few cases that would require regulating, or the mouth require enlarging to let all the teeth in. Is it the baby face we wish to preserve—the nice little baby face? Or do we prefer the face of a man—large and well developed, with jaw enough to compare favorably with the other parts of the face and head? The latter seems to me to be preferable.

How often do we see men, large and well proportioned, with such a delicate little mouth that it is with a great deal of difficulty that we can get one finger to the roof of it. These are the kind of mouths that are not properly attended to when young, and have lost their six year molars.

A lady called at my office a few days since with her little boy, eleven years old. She wished me to look at his teeth, and see what, if any thing, was needed doing to them. I found the right inferior six year molar had been extracted, apparently some years since; the left very badly decayed; both superiors decayed, one considerably. After filling the two above, I took the boy to two of our best dentists, and they both agreed with me that the tooth below should also be preserved. I filled it with Wood's metal. I afterwards had occasion to show the case to another considered first class dentist, whose opinion, judging from his experience in the profession, ought to be good, but in such cases I do not consider it so, for to my great surprise he informed me he should have extracted not only the tooth below but the two above, for the purpose of giving the others room to come in without crowding. He said that when the six year molars showed signs of decay so young, or if the teeth were crowding to any extent, he always extracted them, which operation he usually performed just as the twelve year molars were coming through the gum.

This, gentlemen, is the way the teeth of children are treated by probably two-thirds of the dentists of this country. Is it right? Is it good practice? If so, I must confess I am behind the times, and am in a fair way to remain so. No wonder children look upon dentists as being the most cruel and hard-hearted men in the world, and never want to see them again after their first visit. Those of you who have experienced having teeth extracted, know what pleasant recollections there are connected with the operation, and know something how to sympathize with a child ten or twelve years old, who has it decided by his dentist that he must have his four six year molars extracted. May I be saved from such painful operations.

When I first commenced practice, I extracted these teeth occasionally, when very badly decayed, and also to correct irregularities; but in no case, can I recollect, without feeling a sting of conscience, for I have always felt that these teeth were of more actual importance to every one, not only in preserving the contour of the face, but in that all important operation, mastication, than any other in the mouth. When children lose these teeth so early, they do not masticate their food properly for several years, for the very reason that there is nothing left in the mouth to do it with; hence so many cases of early dyspepsia, that bane of all diseases, which carries so many to an early grave.

Gentlemen, I have not performed the task assigned me as I wish I could have done, nor do I think myself capable of doing the subject justice, for there is a great deal to be said in favor of preserving these teeth, so I will leave it for the consideration of older and more experienced heads.

